# Editorial: Panoramic view of cognitive impairment: interdisciplinary cognitive research

**DOI:** 10.3389/fnins.2024.1453945

**Published:** 2024-07-22

**Authors:** Chong Tian, Song Ge, Lu Ma, Yang Xiao, Fangyi Xu

**Affiliations:** ^1^School of Nursing, Tongji Medical College, Huazhong University of Science and Technology, Wuhan, China; ^2^Department of Natural Sciences, University of Houston-Downtown, Houston, TX, United States; ^3^School of Public Health, Wuhan University, Wuhan, China; ^4^National Key Laboratory of Multispectral Information Intelligent Processing Technology, School of Artificial Intelligence and Automation, Huazhong University of Science and Technology, Wuhan, China; ^5^Brown Cancer Center, Department of Medicine, University of Louisville, Louisville, KY, United States

**Keywords:** cognition, interdisciplinary research, phenomenological description, risk factors, diagnosis

Cognition, information processing activities for individuals to understand the objective world, is a reflection of the real world in the human brain. Cognitive impairment refers to a decline in cognitive functioning of varying degrees, from subjective cognitive impairment to severe dementia. With the accelerated global population aging, the number of people with cognitive impairment is increasing rapidly. Dementia has become a major contributor to the global burden of disease and loss of disability-adjusted life years. Cognitive impairment is a heterogeneous syndrome, the cause of which is still myriad. Although significant advancement in the understanding of the pathology of cognitive impairment has been made in recent decades, the tools and strategies at our disposal to deal with cognitive impairment are still very limited. Currently, no disease-modified treatment has been identified.

Interdisciplinary research refers to the methodology of comprehensive understanding or problem-solving through the intersection between natural science, social science, and other disciplines or the internal intersection between many disciplines within a certain category of science. It is conducive to the overall understanding of problems and identifying effective solutions to complex problems faced by human society. In recent years, studies in many fields other than neurology have inspired our understanding of cognitive impairment, putting forward new insights into its phenomenology and providing many effective prevention or alleviation strategies to delay cognitive decline or manage the symptoms. It is very likely that interdisciplinary research would shed light on our efforts to cope with the challenges brought by the growing epidemic of cognitive impairment.

This research initiative aims to publish cutting-edge, interdisciplinary studies that contribute to a deeper understanding of cognitive impairment. The primary objectives include identifying underlying contributing factors, developing tools for assessment, diagnosis, treatment, or rehabilitation of cognitive impairment, and enhancing daily functioning and quality of life for affected individuals. The overarching goal is to address the escalating epidemic of cognitive impairment. The scope of Research Topics encompasses a wide range, including providing a phenomenological description of the prevalence, risk factors, manifestations, and prognosis of cognitive impairment from diverse disciplinary perspectives. The research also encourages the proposal of novel preventive strategies, the development of innovative assessment and diagnostic methods, and the implementation of multidisciplinary interventions to enhance cognitive functioning and alleviate associated symptoms. The emphasis extends to strategies that aim to preserve daily functioning and improve the overall quality of life for individuals grappling with cognitive impairment. The call welcomes various types of contributions, particularly Original Research Articles, Clinical Trials, Systematic Reviews, Meta-analyses, Reviews, and Mini Reviews. This comprehensive approach seeks to foster advancements in our ability to comprehend, address, and ultimately mitigate the impact of cognitive impairment on individuals and society.

At last, an array of studies addressing cognitive functioning in older adults from different angles were included. Each article presents unique insights, contributing to a nuanced understanding of the multifaceted factors influencing cognitive health. The gathered research spans various dimensions, shedding light on both traditional and emerging aspects of this critical Research Topic.

In exploring the multifaceted landscape of cognitive health in older adults, several studies have delved into diverse aspects of the issue. The study entitled “*Higher blood cotinine level is associated with worse cognitive functioning in non-smoking older adults*” by Fu et al. probed the relationship between secondhand smoke (SHS) exposure, quantified by serum cotinine level, and cognitive functioning in 2,703 non-smoking adults aged 60 and above. It revealed a concerning association, indicating that elevated cotinine levels correlated with diminished cognitive scores, affecting memory, fluency, and overall cognitive function negatively. This study underscores the imperative of reducing SHS exposure in older adults to safeguard cognitive health, advocating for targeted public health interventions. Moving beyond conventional factors, the article “*Sensory impairment and cognitive decline among older adults: An analysis of mediation and moderation effects of loneliness*” by Ge et al. unraveled intricate connections between sensory impairment, loneliness, and cognitive decline. Emphasizing the importance of addressing social and psychological wellbeing in older adults with sensory impairment, the study highlighted that visual impairment coupled with heightened loneliness may exert a more detrimental impact on cognitive function than visual impairment alone. This study shed light on the nuanced interplay between sensory impairment, loneliness, and cognitive decline in aging populations. In another study entitled “*Impact of hearing loss on cognitive function in community-dwelling older adults: serial mediation of self-rated health and depressive anxiety symptoms*,” by Chen et al. it was found that hearing loss not only directly affected cognitive function negatively but also had an indirect impact through self-rated health and depressive anxiety symptoms. Serial mediation analysis indicated that the total indirect effect of self-rated health and depressive anxiety symptoms accounted for 52.04% of the total effect of the model. This underscores the importance of enhancing self-rated health and promoting good mental health to potentially delay the cognitive decline associated with hearing loss in older adults. Risk factors for agitation in home-cared older adults with dementia are dissected through evidence gleaned from a substantial elder population in East China. The article “*Risk factors for agitation in home-cared older adults with dementia: evidence from 640 elders in East China*” by Liu et al. revealed that 42.8% of the sample exhibits agitated behaviors. Risk factors include basic health issues such as activities of daily living (ADL), family support issues measured by the Zarit Burden Interview (ZBI) scale and Family APGAR Questionnaire (APGAR), and behavioral awareness issues like falls and scalds. Older adults with severe ADL disorders, a high ZBI score, severe APGAR disorders, and a history of falls or scalds are more likely to exhibit agitated behaviors.

The Research Topic unfolded the intricate association between serum biomarkers and cognitive health, touching upon lead levels, globulin, cystatin C, and cardiovascular risk markers. The study “*Concurrent serum lead levels and cognitive function in older adults*” by Deng et al. investigated the correlation between serum lead levels and cognitive function in older adults from the National Health and Nutrition Examination Survey (NHANES) 2011–2013, showed that serum lead concentration was not associated with cognitive performance in older adults. The study “*Association between serum globulin and cognitive impairment in older American adults*” by Huang et al. revealed a non-linear association between serum globulin levels and cognitive impairment, with inflection points identified for specific tests. Elevated serum globulin was linked to increased cognitive impairment, emphasizing a potential threshold effect. The findings highlighted the complex, non-linear nature of the association between serum globulin and cognitive function. The cross-sectional study entitled “*Association between serum cystatin C level and cognition in older adults: a cross-sectional analysis*” by Wang S. et al. investigated the relationship between serum Cystatin C levels and cognition in U.S. older adults. Revealing that higher serum Cystatin C levels were independently associated with lower scores in processing speed, sustained attention, and working memory. This research identified a potential link between kidney function, as indicated by Cystatin C levels, and specific cognitive domains.

Similarly, the article titled “*Association of Life's Simple 7 with mild cognitive impairment in community-dwelling older adults in China: a cross-sectional study*,” by Yang et al. a cross-sectional study in Chinese community-dwelling older adults, investigated the association between Life's Simple 7 (LS7), a cardiovascular health metric, and mild cognitive impairment (MCI). The study found a significant association between MCI and both the overall LS7 score and the biological score, even after adjusting for demographic and cardiovascular factors, suggesting that adhering to LS7 guidelines may have a positive impact on preventing MCI in the community, implementing LS7 guidelines could serve as a valuable strategy for community-based interventions to safeguard cognitive health in older adults. This research highlighted the potential link between cardiovascular health and cognitive wellbeing. These studies underscored the importance of holistic approaches for cognitive wellbeing in older individuals. A population-based cross-sectional study entitled “*The neutrophil-to-lymphocyte ratio is associated with mild cognitive impairment in community-dwelling older women aged over 70 years: a population-based cross-sectional study*” by Li et al. revealed that a higher NLR was an independent risk factor for MCI in women older than 70, with an odds ratio of 1.28. The review “*The role of pyroptosis in cognitive impairment*” by Yang and Tang explored the role of pyroptosis, a pro-inflammatory form of programmed cell death, in the occurrence and progression of cognitive impairment. The exploration of potential therapeutic implications of targeting pyroptosis offers valuable insights for future cognitive impairment research. Understanding the link between pyroptosis and cognitive decline may open new avenues for developing effective therapeutic interventions in the field.

The complex interplay between exercise, sleep, and cognitive health in older populations was studied in several studies. The study “*Threshold effects of the relationship between physical exercise and cognitive function in the short-sleep elder population*” by You et al. indicated a positive association between exercise volume and cognitive scores in the Animal Fluency and Digit Symbol Substitution tests. However, a two-piecewise linear regression model revealed a threshold effect, suggesting that cognitive benefits did not consistently increase with higher exercise volumes for short-sleep elders. The study challenges existing knowledge by proposing that cognitive performance in the short-sleep elderly group can be maintained with no more than 800 MET-min/week of physical exercise. The article “*Association between sedentary behavior and risk of cognitive decline or mild cognitive impairment among the elderly: a systematic review and meta-analysis*” by Cai et al. investigated the association between sedentary behavior (SB) and the risk of cognitive decline (CD) or mild cognitive impairment (MCI) in the elderly, revealing a significant positive association between SB and the increased risk of CD (OR = 1.69) or MCI (OR = 1.34) in the elderly. Subgroup analyses considering comorbidity, lifestyle, family structure, publication year, and region demonstrated statistical differences between groups, contributing to the understanding of heterogeneity sources. The findings support the notion that reducing sedentary behavior may contribute to maintaining cognitive health in the elderly and provide valuable evidence for clinicians and policymakers to promote healthy behaviors in the elderly population.

Several studies contribute valuable insights into the prevalence, diagnosis, and perception of cognitive health and prevention. The article “*Evidence from a meta-analysis and systematic review reveals the global prevalence of mild cognitive impairment*” by Song W-x. et al. aimed to determine the global prevalence of mild cognitive impairment (MCI). Analyzing 233 studies with 676,974 individuals aged above 50, the study found an overall global MCI prevalence of 19.7% and an increasing trend in global MCI prevalence, particularly after 2019, with a significant rise to 32.1%. Additionally, MCI prevalence was higher in hospitals (34.0%) compared to nursing homes (22.6%) and communities (17.9%), emphasizing the need for further attention and resource allocation to address MCI in at-risk populations. The study “*Perception and knowledge of dementia prevention and its associated socio-demographic factors in China: a community-based cross-sectional study*” by Song D. et al. extends to the realm of societal perceptions and knowledge surrounding dementia prevention. The authors found that only 32.4% of Chinese adults aged over 40 believed dementia was preventable. The findings underscore significant disparities in public knowledge and highlight the need for comprehensive educational programs targeting all age groups. The exploration offered a glimpse into the socio-demographic factors shaping these perspectives in China and emphasized that specific attention should be given to individuals with lower income and education levels to enhance their access to dementia prevention and management resources. Practical diagnostic tools are discussed. The authors of the article “*A dual-task gait test detects mild cognitive impairment with a specificity of 91.2%*” by Wang Y. et al. aimed to develop a convenient method for detecting mild cognitive impairment (MCI) in the community. Utilizing a novel dual-task gait test, in which participants went through a gait test while identifying animals in pictures (AniP-DT gait test), participants with MCI could be identified through the gait performance with a specificity of 91.2%, highlighting its potential as an easy and reliable screening tool for older adults in the community setting. The study emphasizes the significance of integrating dual-task gait assessments for community-based cognitive screening, offering a practical approach to detecting cognitive decline in aging populations. The article “*Machine vision-based gait scan method for identifying cognitive impairment in older adults*” by Qin et al. introduced a machine vision-based gait scan method, DO-GaitPart, for identifying cognitive impairment in older adults. Employing a dataset labeled with cognitive performance scores, DO-GaitPart outperforms other methods in gait recognition tasks and achieved a promising ROCAUC of 0.876 in cognitive state classification, offering a valuable tool for early detection and management of age-related cognitive impairments.

Exploring therapeutic interventions for mild cognitive impairment (MCI), two comprehensive reviews offer valuable insights into potential avenues for enhancing cognitive wellbeing. The systematic review and meta-analysis “*Donepezil combined with traditional Chinese medicine has promising efficacy on mild cognitive impairment: a systematic review and meta-analysis*” by Yu et al. evaluated the efficacy and safety of combining donepezil and traditional Chinese medicine (TCM) for treating mild cognitive impairment (MCI), revealing that the combination significantly improved cognitive function [Montreal Cognitive Assessment [MoCA] score] and activities of daily living (Barthel Index score) compared to donepezil alone. However, subgroup analysis suggested that MoCA scores did not significantly increase in MCI patients resulting from cerebrovascular diseases. This research provided insights into potential therapeutic approaches for MCI by combining Western medication with traditional Chinese medicine, although further high-quality studies are still needed to confirm these promising findings. In a parallel exploration, the review “*Effects of exercise therapy on patients with poststroke cognitive impairment: a systematic review and meta-analysis*” by Zhang et al. revealed that exercise therapy significantly improved cognitive function, motor function, and activities of daily living in individuals with poststroke cognitive impairment. The findings underscore the therapeutic benefits of exercise in enhancing patients' both cognitive and physical performance, emphasizing the importance of medical practitioners prioritizing the active use of exercise therapy to enhance the cognitive function, motor skills, and daily living activities in stroke survivors. Together, these reviews contribute to a broader understanding of therapeutic strategies for cognitive impairment, emphasizing the potential of combined interventions and the pivotal role of exercise in promoting cognitive and physical wellbeing.

Collectively, this Research Topic offers a panoramic view of cognitive functioning in aging population, presenting a synthesis of evidence and insights from diverse angles. Bringing together these studies serves as a valuable resource for researchers, healthcare professionals, and policymakers seeking a comprehensive understanding of factors influencing cognitive health in older adults.

## Author contributions

CT: Writing – review & editing, Writing – original draft, Funding acquisition, Conceptualization. SG: Writing – review & editing, Resources. LM: Writing – review & editing. YX: Writing – review & editing. FX: Writing – review & editing.

